# Systemic Complement Activation in Age-Related Macular Degeneration

**DOI:** 10.1371/journal.pone.0002593

**Published:** 2008-07-02

**Authors:** Hendrik P. N. Scholl, Peter Charbel Issa, Maja Walier, Stefanie Janzer, Beatrix Pollok-Kopp, Florian Börncke, Lars G. Fritsche, Ngaihang V. Chong, Rolf Fimmers, Thomas Wienker, Frank G. Holz, Bernhard H. F. Weber, Martin Oppermann

**Affiliations:** 1 Department of Ophthalmology, University of Bonn, Bonn, Germany; 2 Institute of Medical Biometry, Informatics and Epidemiology, University of Bonn, Bonn, Germany; 3 Department of Cellular and Molecular Immunology, University of Göttingen, Göttingen, Germany; 4 Institute of Human Genetics, University of Regensburg, Regensburg, Germany; 5 Oxford Eye Hospital, University of Oxford, Oxford, United Kingdom; Ohio State University Medical Center, United States of America

## Abstract

Dysregulation of the alternative pathway (AP) of complement cascade has been implicated in the pathogenesis of age-related macular degeneration (AMD), the leading cause of blindness in the elderly. To further test the hypothesis that defective control of complement activation underlies AMD, parameters of complement activation in blood plasma were determined together with disease-associated genetic markers in AMD patients. Plasma concentrations of activation products C3d, Ba, C3a, C5a, SC5b-9, substrate proteins C3, C4, factor B and regulators factor H and factor D were quantified in patients (n = 112) and controls (n = 67). Subjects were analyzed for single nucleotide polymorphisms in factor H (*CFH*), factor B-C2 (*BF-C2*) and complement C3 (*C3*) genes which were previously found to be associated with AMD. All activation products, especially markers of chronic complement activation Ba and C3d (p<0.001), were significantly elevated in AMD patients compared to controls. Similar alterations were observed in factor D, but not in C3, C4 or factor H. Logistic regression analysis revealed better discriminative accuracy of a model that is based only on complement activation markers Ba, C3d and factor D compared to a model based on genetic markers of the complement system within our study population. In both the controls' and AMD patients' group, the protein markers of complement activation were correlated with *CFH* haplotypes.

This study is the first to show systemic complement activation in AMD patients. This suggests that AMD is a systemic disease with local disease manifestation at the ageing macula. Furthermore, the data provide evidence for an association of systemic activation of the alternative complement pathway with genetic variants of *CFH* that were previously linked to AMD susceptibility.

## Introduction

Age-related macular degeneration (AMD) is the leading cause of blindness in all populations of European origin [1]. The pathogenesis of AMD is not well understood with both genetic and environmental factors known to influence susceptibility to this disease [2]. A hallmark of early disease are drusen, lipoproteinaceous deposits which accumulate in the space between the retinal pigment epithelium (RPE) and Bruch's membrane. Late AMD is broadly classified into two clinical forms, a dry form with geographic atrophy (GA), characterized by loss of RPE and outer neurosensory retinal cells, and a wet form with choroidal neovascularization (CNV). An estimated 1.75 million US Americans suffer from late AMD and another 7.3 million have signs of early AMD putting them at substantial risk for vision loss from this devastating disease [1].

Studies on the molecular composition of drusen have implicated inflammation, and particularly local activation of the alternative pathway (AP) of the complement cascade in the retina, in the pathogenesis of AMD [3]. Furthermore, strong evidence for a role of complement in this disease derives from an independent line of research which showed that variants in the complement factor H (*CFH*) gene are significantly associated with an increased risk for AMD in Caucasian populations [4]–[7]. These genetic studies were recently extended by the observation that polymorphisms in other complement genes, notably those coding for factor B-complement component C2 (*BF-C2*) and complement C3 (*C3*), are also associated with AMD [8]–[11]. Multiple haplotypes in the *CFH* and *BF* genes appear to modulate the AMD disease risk and both disease-predisposing and protective gene variants were identified [7], [8], [12].

**Figure 1:**
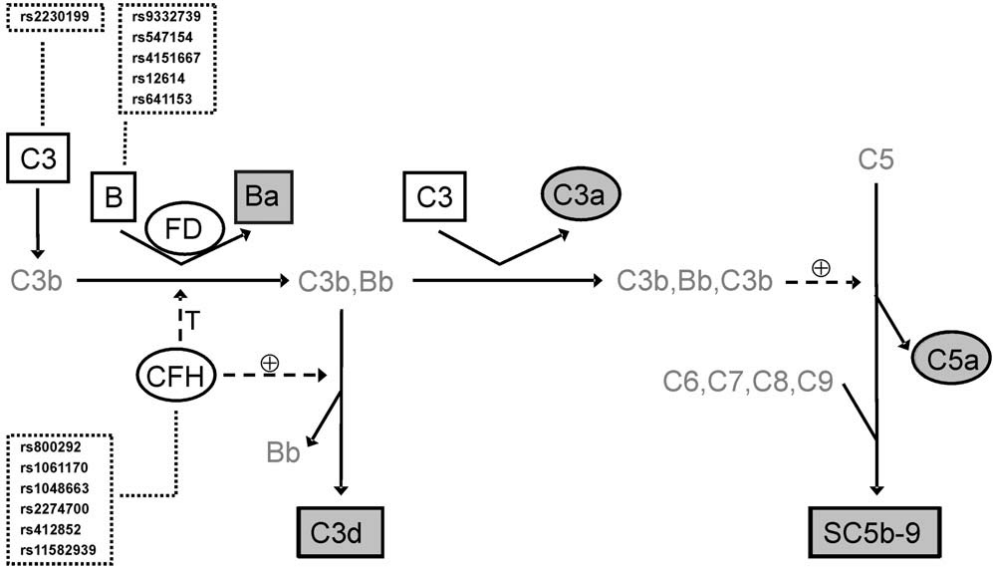
The Alternative Pathway of Complement: Polymorphic Variants and Complement Proteins under Study. Complement gene SNPs (boxed with dotted lines) and protein plasma concentrations (boxed with solid lines) were determined in all AMD patients and controls. C3, C4 and factor B are substrates (open rectangles), factor H and factor D are regulators (open ellipses), Ba, C3d and SC5b-9 are markers of chronic activation (filled rectangles), and C3a and C5a are markers of acute activation (filled ellipses) of the alternative complement pathway.

Activation of the AP of complement on cellular surfaces results from the failure to downregulate the spontaneous low-level activation of C3. Factor H, the major inhibitor of the AP of complement activation in the fluid-phase, binds to host cells and inhibits complement activation by its ability to interfere with the formation and activity of the alternative C3 convertase, C3bBb. It accelerates the decay of this convertase and acts as a cofactor for the factor I-mediated proteolytic inactivation of C3b into iC3b and C3dg [13]. In the absence of factor H, C3b binds factor B, allowing its cleavage by the serine protease factor D to yield the fragments Ba and Bb. Eventually, this results in the formation of the alternative C5 convertase and assembly of terminal complement components into the C5b-9 membrane-attack complex (Fig. 1). Factor H is among the most abundant complement proteins in serum, synthesized predominantly in the liver, but to a lesser extent also locally in the eye by RPE cells [14]. Within the superfamily of functionally and structurally related cofactors for Factor I-mediated C3b degradation (factor H, CR1, CR2, MCP) and for the acceleration of the decay of the C3 convertases (factor H, CR1, DAF), factor H is the main regulator which acts as a soluble protein. This possibly explains systemic consequences which could result from polymorphic variation of this protein.

Subtle differences in plasma concentrations or functional activities of negative (factors H and I) or positive (factor D) complement regulatory proteins, as well as differences in the substrates factor B and C3, could have a significant impact on the magnitude of local complement activation in response to a given stimulus. Consequently, low-level activation of the AP of complement may result in the local release of pro-inflammatory and angiogenic mediators as well as tissue damage in the retina which eventually may lead to manifest disease. Based on the hypothesis that defective control of complement activation leads to the release of complement cleavage products which are detectable in the circulation, we performed a comprehensive investigation of AP of complement protein plasma concentrations in a cohort of AMD patients and controls. The findings were correlated with polymorphisms in the *CFH*, *BF-C2*, and *C3* genes.

## Results

The study population included 112 AMD patients and 67 control subjects of similar age, gender and smoking habits which showed no signs of macular disease (Table 1). Plasma concentrations of complement proteins in the study population are shown in Table 2. All complement activation products, and most prominently markers of chronic complement activation C3d and Ba (p<0.001), were significantly elevated in AMD patients as compared to controls. The small C3a and C5a anaphylatoxins, which are rapidly eliminated from blood plasma, and SC5b-9, which is generated downstream of C3 and factor B activation, were also detectable at higher levels, although these differences were less pronounced. Complement activation cannot be attributed to different plasma concentrations of factor H, nor of C3 or C4, which were found to be very similar in both study groups. Factor D plasma levels were significantly (p<0.001) higher in the patients' group.

**Table 1:** Clinical and Demographic Characteristics of the Study Population.

Variable	Controls	AMD patients
	(N = 67)	(N = 112)
**Disease status - no. (%) ** *		
Early AMD (Drusen)	-	9 (8%)
Choroidal neovascularization	-	78 (70%)
Geographic atrophy	-	25 (22%)
**Gender - no. (%) ** **		
Male	37 (55%)	41 (37%)
Female	30 (45%)	71 (63%)
**Mean age - years (SD; range)** ***	70.1 (6.0; 60–86)	75.6 (6.6; 59–94)
**Smoking history – no. (%)**		
Never smoked	35 (52%)	59 (53%)
Ex-smoker	26 (39%)	43 (38%)
Current smoker	6 (9%)	10 (9%)

* AMD patients were categorized into mutually exclusive groups: All subjects classified as “early AMD” (n = 9) had extensive intermediate and/or large drusen in at least one eye and all qualified as category III of the Age-Related Eye Disease Study group. Patients with GA in both eyes or GA in one eye in the absence of CNV in the fellow eye were classified as “GA” (n = 25). Nineteen of these had subfoveal GA in at least one eye; the remaining six patients exclusively showed extrafoveal GA. If a CNV due to AMD was present in at least one eye, patients were classified as “CNV” (n = 78). Subjects with changes such as many hard drusen and/or pigmentary alterations in both eyes were excluded as these changes may refer to normal aging processes not necessarily linked to AMD.

** Difference between cases and controls, p = 0.02; ^***^ p<0.001.

**Table 2:** Plasma Concentrations of Complement Proteins in AMD Patients and Controls.

Complement protein *	Units	Controls		AMD patients		p **
		Median	5^th^, 95^th^ Ptcl.	Median	5^th^, 95^th^ Ptcl.	
C3	[mg/ml]	1.18	0.85–1.48	1.12	0.89–1.53	0.85
C4	[mg/ml]	0.24	0.15–0.34	0.23	0.15–0.38	1.0
Factor B	[µg/ml]	642	378–1354	803	497–1489	0.02
Factor H	[µg/ml]	515	365–711	546	396–758	0.21
Factor D	[µg/ml]	0.95	0.50–1.65	1.26	0.69–2.30	<0.001
C3a	[ng/ml]	14.3	10.6–21.2	15.5	11.2–24.1	0.03
C5a	[ng/ml]	1.67	0.66–2.32	1.85	0.78–2.66	0.04
Ba	[µg/ml]	1.09	0.60–1.71	1.33	0.90–2.09	<0.001
C3d	[µg/ml]	46.9	32.2–68.5	55.2	35.7–94.1	<0.001
SC5b-9	[units]	159	90–710	188	107–777	0.01

* Complement proteins in this table are arranged in groups of substrates (C3, C4, factor B), regulators (factor H, factor D), markers of acute (C3a, C5a) and chronic activation (Ba, C3d, SC5b-9) of the alternative pathway of complement (Fig. 1).

** Wilcoxon rank-sum test; corrected for multiple testing by Bonferroni-Holm procedure.

An analysis of phenotypic subgroups revealed that only the C3d concentration in plasma was differently distributed (ANOVA, uncorrected p<0.001), the CNV subgroup exhibiting lowest C3d levels compared to patients with geographic atrophy and patients with early AMD. The degree to which this finding can be generalized is limited due to the small number of patients which were studied within the different phenotypic subgroups.

To determine whether AP of complement activation in AMD patients is related to complement gene polymorphisms previously associated with the disease, all probands were genotyped for six SNPs in the *CFH* gene, five in *BF-C2*, and one polymorphism in *C3* (Fig. 1 and Table S1). In accordance with previous reports [9]–[12], four markers in *CFH* (I62V, Y402H, A473A, IVS 15), two markers in *BF-C2* (IVS 10, R32Q) and R102G in *C3* were significantly associated with AMD. Haplotype analysis of the SNPs in *CFH* revealed one risk haplotype, GCGGGC, and two protective haplotypes, ATGAAC and GTGAAC (Table S2). For the definition of the *CFH* risk haplotype, the Y402H polymorphism was sufficient. The risk haplotype was observed in 57% of cases but only 36% of controls, whereas the protective haplotypes were observed in 12% and 8% of cases and in 23% and 18% of controls, respectively. The distribution of haplotypes was different between cases and controls for *CFH* (p<0.001), but not for *BF-C2* (p = 0.14).

**Figure 2:**
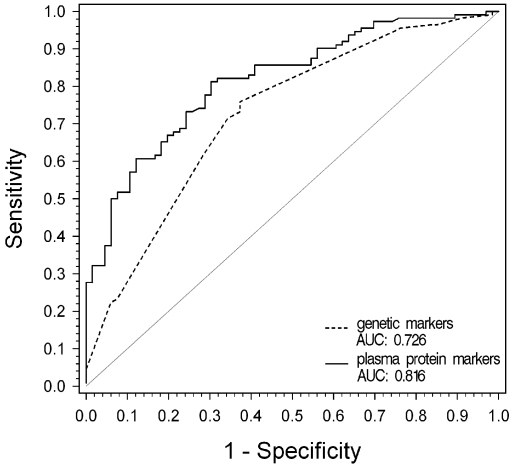
Receiver Operating Characteristic (ROC) Curves for the Discriminative Capability of Genetic and Protein markers of the Complement System. Receiver operating characteristic curves for genetic markers (dotted line; A473A of *CFH*, IVS 10 of *BF-C2* and R102G of *C3*) and complement protein markers (solid line; Ba, C3d, and factor D) are shown. AUC = Area under ROC curve.

Logistic regression analysis yielded significance for six variables including three protein markers and three genetic markers. A subsequent stepwise logistic regression analysis including exclusively genetic markers revealed the three genetic markers A473A at *CFH*, IVS 10 at *BF-C2*, and R102G at *C3* to be the best genetic predictors of risk for AMD with a high accuracy for discrimination and an area under the curve (AUC) value of 0.726 (Fig. 2). A stepwise logistic regression analysis including only protein markers resulted in a model with the three markers Ba, C3d and factor D. The corresponding ROC curve showed an even better discriminatory accuracy (AUC = 0.816; p = 0.05). When both genetic and protein markers were included into the stepwise regression procedure, the resulting model revealed only a marginally better fit (AUC = 0.850).

To detect a direct link between genetic variants at *CFH* and protein markers of AP of complement activation, plasma levels were compared between carriers of the *CFH* risk haplotype and carriers of the protective haplotypes, both within the controls' and the AMD patients' group (Table 3). Within both subject groups (with the exception of C5a in the patients' group), carriers of the *CFH* risk haplotype showed consistently higher complement activation parameters than those carrying the protective haplotypes. A correlation between genetic variants in *BF-C2* or *C3* and complement protein levels was not observed.

**Table 3:** Plasma Concentrations (mean±S.E.M.) of Complement Proteins in Carriers of Risk-Conferring and Protective Haplotypes of the *CFH* Gene.

Complement protein	Units	Controls			AMD patients		
		All	Risk haplotype	Protective haplotypes	All	Risk haplotype	Protective haplotypes
		(n = 67)	(n = 17)	(n = 23)	(n = 112)	(n = 67)	(n = 16)
Ba	[µg/ml]	1.11±0.04	1.21±0.25	1.04±0.08	1.41±0.04	1.46±0.05	1.27±0.11
C3d	[µg/ml]	48.1±1.40	50.9±3.71	45.4±2.34	59.1±1.80	60.6±2.11	52.7±4.88
C3a	[ng/ml]	14.7±0.38	15.3±0.70	14.0±0.62	16.4±0.37	16.6±0.53	15.3±0.89
C5a	[ng/ml]	1.55±0.06	1.64±0.12	1.46±0.11	1.77±0.06	1.72±0.07	1.86±0.16
Factor D	[µg/ml]	0.98±0.04	1.04±0.05	0.85±0.07	1.35±0.05	1.38±0.06	1.26±0.12

## Discussion

A low-key but persistent inflammatory process which involves activation of the complement system has been proposed to underlie the sight-threatening manifestations in AMD. In support of this hypothesis, various inflammatory mediators including complement proteins, their activation products and regulators have been identified in retinal deposits of AMD patients. In particular, complement components C3 and C5, the membrane attack complex C5b-9 and factor H, the main regulator of alternative pathway activation, are constituents of drusen in AMD patients [16]. Further evidence for a role of complement in this disease derives from two recent studies which showed that laser-induced CNV, an accelerated model of neovascular AMD in mice, requires intact C3 and cellular C3aR/C5aR receptors [17], [18]. Thus, uncontrolled activation of the AP of complement within the central retina may play a major role in the pathophysiology of AMD through its ability to promote local tissue damage and angiogenesis [17].

In the present study we show that several parameters which reflect systemic complement activation are significantly elevated in the circulation of AMD patients as compared to controls. We exercised great caution to avoid in vitro activation of complement. As illustrated in a recent study on complement activation in AMD patients, erroneously elevated concentrations of complement fragments are detected, when heparin- rather than EDTA-anticoagulated blood was used for analysis [19]. Although we concur with the overall conclusions from this particular study, the presented data are largely artifactual and result from ex vivo complement activation in the heparinized blood tubes [40], [41]. As previous studies pointed to a role of complement activation in AMD we focused our analysis on the quantification of the major breakdown products of C3 and factor B, i.e. C3a, C3d, and Ba. Among these, Ba and C3d are particularly well suited as markers of chronic AP of complement activation at low levels, since their respective half-lives in-vivo are in the order of several hours rather than minutes [20]. Since complement turnover in vivo is further determined by biosynthetic rates of the precursor proteins as well as by concentrations of complement control proteins, we determined plasma levels of the corresponding precursor proteins C3 and factor B, and the main positive and negative regulators of the AP of complement, factors D and H. C5a and SC5b-9 were quantified as markers of terminal pathway of complement activation which are generated downstream of C3 and factor B. The simultaneous quantification of the main cleavage products, substrates and of control proteins of the AP of complement is a unique feature of this study and allowed to precisely document the state of complement activation in all patients.

Our notion of enhanced systemic complement activation in AMD is mainly based on the finding that in patients all complement activation products determined in this study were elevated as compared to controls. This difference was most strikingly observed with regard to Ba and C3d, two sensitive markers of chronic AP of complement activation in-vivo. In contrast, the complement proteins C3, C4 and factor H did not significantly differ between the two groups. We also observed elevated levels of factors B and D in AMD patients and this could possibly be due to an acute phase response-mediated upregulation of factor B or by polymorphic variation in the *FD* gene which may affect factor D plasma levels. Upregulation of these two positive regulators may further contribute to enhanced AP of complement activation, however, subtle changes in factors B and D alone cannot explain the enhanced turnover of complement substrates [21]. While Ba and C3d concentrations in AMD patients were only modestly elevated by a factor of 1.2 to 1.3 compared to controls, C3d levels in the plasma of patients with rheumatoid arthritis are also increased to a similar degree [22]. Since local C3d concentrations in synovial fluids from these patients are much higher than in plasma [23], a similar gradient between sites of local complement activation and blood plasma may also exist in AMD.

Our results further demonstrate that a combination of complement activation markers can be used to most reliably discriminate AMD patients from controls in our study population. The discriminatory ability of these complement proteins (AUC = 0.816) appears superior or at least similar to the discriminatory ability of genetic markers of complement genes for the prediction of AMD as determined in this and other similarly designed studies [24]. Because the protein markers are closer to the presumed mechanisms of AMD pathogenesis, this finding appears plausible. Prospective studies will be required to determine whether plasma concentrations of complement proteins could be useful as markers of AMD at less advanced stages, either alone or in combination with genetic markers. Since this study was limited to genes and proteins of the alternative pathway of complement, a general conclusion on the superiority of protein markers compared to genetic markers is not possible. Such a conclusion may be derived from an even more comprehensive study including additional genetic and protein markers. Since the *LOC387715/HTRA1/ARMS2* locus has been shown to be of similar significance as *CFH* [25], polymorphisms within *ARMS2* would have to be considered. Recently, the discriminative accuracy of testing polymorphisms in *CFH* and *BF-C2* together with *ARMS2* for the prediction of AMD was found to be 80% [24], which is similar to the score we found for testing protein markers of the alternative pathway of complement.

Our study has linked elevated plasma concentrations of AP of complement activation products in AMD patients to polymorphic variations in the *CFH* gene which codes for the main regulator of the AP of complement activation in the fluid phase and on cell surfaces [13]. We observed within both study groups that individuals who carry the AMD-associated *CFH* risk haplotype had higher plasma concentrations of complement activation products, and conversely that protective *CFH* haplotypes were associated with lower levels of activation products (p = 0.05, MANOVA).

In particular, the association between complement activation and the *CFH* risk haplotype which includes Y402H, a non-synonymous SNP in *CFH* which leads to a tyrosine to histidine exchange at position 402, is biologically plausible. Several recent studies have shown that the His402 variant binds less well to heparin, C reactive protein and RPE cell surfaces [14], [26], [27]. While the consequences of defective factor H function are most likely not restricted to the eye, but result in inappropriate complement control at other cell surfaces throughout the body, the retina appears to be especially sensitive towards the effects of complement activation.

Our data suggest that AMD indeed is a systemic disease with local disease manifestation at the ageing macula. Such local manifestation of systemic pathophysiology is not without precedence. An example are mutations in the PRPF3 gene causing a tissue-specific phenotype of autosomal dominant retinitis pigmentosa although PRPF3 is an element of the ubiquitously expressed RNA splicing machinery [28]. In addition, the macula is more easily damaged than the retinal periphery, e.g. because it exhibits decreased thickness and integrity of the elastic layer of Bruch's membrane [29]. The hypothesis of AMD being a systemic disease certainly raises the possibility of other organ manifestations that have so far not been detected. In support of this hypothesis, patients with membranoproliferative glomerulonephritis type II (MPGN II) and systemic complement activation develop retinal deposits at early ages which resemble drusen in AMD patients [30]. Since MPGN II patients benefit from substitution therapy with intact complement control proteins, local or systemic administration of AP of complement inhibitors may be considered as a future therapeutic option in AMD.

## Materials and Methods

### Cases and Controls

112 patients with a clinical diagnosis of AMD and 67 control subjects were included in the study. Absence of AMD, early and late AMD was defined according to the criteria of the ARM Epidemiological Study Group [31]. Exclusion criteria included age below 55 years; any evidence of retinal disease (in the control group) with the exception of AMD (in the AMD group) ascertained by history, clinical examination, digital fundus photography and grading of fundus images (see below); any systemic disease known to affect the complement system (e.g. rheumatoid arthritis) ascertained by a standardized case report form (CRF) derived from the multicenter FAM-Study [32] and abnormal renal clearance (ascertained by creatinine and cystatin C values). In the recruitment of the control subjects particular attention was paid to match for smoking habits (ascertained by the CRF), since smoking has been shown to be by far the most significant environmental risk factor for AMD. Moreover, earlier studies have suggested an effect of smoking on factor H blood levels [13]. As a result, the control group was of similar age and gender, and exhibited identical smoking habits; minor differences in age and gender of the two groups were regarded irrelevant for complement protein concentrations [41]. All subjects were of Caucasian descent and were recruited within the same time period from the Department of Ophthalmology, University of Bonn between January and October 2006. Control subjects did not have any signs of AMD, especially no early changes such as many small drusen, intermediate or large drusen. Informed consent was obtained from all subjects. The research protocol was in keeping with the provisions of the Declaration of Helsinki, and approval was obtained from the institutional ethics committee. Digital fundus photographs were obtained from all participants. In patients with CNV, optical coherence tomography and fluorescein angiography were performed. Fundus autofluorescence imaging was performed in patients with GA. All fundus images were evaluated separately by two independent readers (HPNS and PCI); digital fundus images were graded according to the classification system of the International ARM Epidemiological Study Group [31], [33], [34]. Clinical characteristics and demographic data of the study populations are provided in Table 1.

### Blood samples

Venous blood was collected from all subjects into tubes containing dipotassium EDTA at a final concentration of 8 mM. The plasma was separated from the blood cells by centrifugation (20 min/1000× g) within 3 hours after venipuncture and frozen in aliquots at −80°C until analysis. One subject with elevated complement levels due to mild chronic renal failure was excluded from the analysis of factor D and Ba since the catabolism of these two complement proteins is determined by the glomerular filtration rate [21]. All other subjects had normal creatinine and cystatin C values; both subject groups were not significantly different for these variables.

### Analysis of complement proteins

Assays for the quantitation of complement components factor B, Ba, C3a, C3d, C5a, SC5b-9, factor D and factor H have been developed previously [35]–[37]. All complement activation assays were based on monoclonal antibodies with specificities for activation-induced neoepitopes present on the different complement split products which are absent from the respective native precursor proteins. In the case of the ‘C3d’-assay, a capture mAb (I3/15) was used which reacts with a common neoepitope on C3b, iC3b, and C3dg, in combination with a polyclonal rabbit anti-C3d as the detecting antibody [35]. Plasma concentrations of C3 and C4 were determined by rate nephelometry. All patients and control samples were handled identically and analyzed simultaneously in order to ensure stable assay conditions. As reported before [36], the inter-assay coefficient of variation of ELISA procedures was below 10%. The intra-individual stability of complement plasma levels was assessed in a subset of AMD patients (n = 14). From these patients, a second ETDA plasma sample was obtained 12 months after the first venipuncture. Within this period of time, Ba, C3d and factor D values varied by less than 15% compared to the initial values.

### Genotyping

Genomic DNA was extracted from peripheral blood leukocytes following established protocols. Genotyping was done by TaqMan SNP Genotyping or by direct sequencing of SNPs. TaqMan Pre-Designed SNP Genotyping Assays (Applied Biosystems, Foster City, U.S.A.) were performed according to the manufacturer's instructions and were analyzed with a 7900HT Fast Real-Time PCR System (Applied Biosystems). Direct sequencing was performed with the Big Dye Terminator Cycle Sequencing Kit Version 1.1 (Applied Biosystems) according to the manufacturer's instructions. Reactions were analyzed with an ABI Prism Model 3130xl Sequencer (Applied Biosystems). Individual genotypes that were ambiguous or missing were reanalyzed resulting in a call-rate of 100% for all SNPs tested. SNP-IDs for three complement gene loci *CFH*, *BF-C2*, and *C3* are indicated in Fig. 1.

### Statistical Analysis

Based on data of assay variability of the key proteins of chronic AP of complement activation (C3d, Ba, SC5b-9) derived from our laboratory [35], [36], the study was designed to detect a difference of 2/3 of a standard deviation between cases and controls with a power of at least 90%, with a two sided test at a level of α = 0.05/3. The level had been chosen to account for the fact that three biochemical markers had to be compared. The recruitment was planned to be imbalanced with a case control ratio of 2∶1 resulting in 120 cases and 60 controls to be included.

Allele and genotype frequencies were determined. All markers were in Hardy-Weinberg equilibrium (all p>0.20). All allele frequencies were within the ranges reported in the Entrez SNP database (www.ncbi.nih.gov) and in previous publications [4]–[12]. To test for genetic association, genotype frequencies were compared between cases and controls by Armitage's trend test. A retrospective power analysis based on the empirical values for allele frequencies and relative risks found in our data was performed [38]. Results are reported in Table S3. Haplotypes for the markers in *CFH* and *BF-C2* were estimated using FAMHAP [39]. The difference in distribution of haplotypes was tested by likelihood ratio test.

Stepwise logistic regression analysis was used to explore models to predict the risk for AMD depending on genetic markers and complement protein markers and to gain more insight into the relevance of these risk parameters in relation to one another. Results were visualized by receiver operating characteristic (ROC) curves for the scores resulting from the logistic regression. ROC curves were compared using the method proposed by DeLong et al [15]. Multivariate analysis of variance (MANOVA) was applied to the joint distribution of complement activation markers to verify the observation of simultaneously increased values depending on *CFH* haplotypes and disease status. Data were handled in SAS (SAS software package for Windows, version 9.1.; SAS Institute Inc., Cary, NC, USA; http://www.sas.com).

## Supporting Information

Table S1 Genotypes
(0.09 MB PDF)

Table S2 Haplotypes
(0.08 MB PDF)

Table S3 Retrospective Power Analysis
(0.17 MB PDF)
