# Correction: Systemic Complement Activation in Age-Related Macular Degeneration

**DOI:** 10.1371/annotation/32b9bc31-ed6d-4d31-9ce0-480407017bad

**Published:** 2008-07-11

**Authors:** Hendrik P. N. Scholl, Peter Charbel Issa, Maja Walier, Stefanie Janzer, Beatrix Pollok-Kopp, Florian Börncke, Lars G. Fritsche, Ngaihang V. Chong, Rolf Fimmers, Thomas Wienker, Frank G. Holz, Bernhard H. F. Weber, Martin Oppermann

The second author's name appears incorrectly in the citation. The correct citation should read: Scholl HPN, Charbel Issa P, Walier M, Janzer S, Pollok-Kopp B, et al. (2008) Systemic Complement Activation in Age-Related Macular Degeneration. PLoS ONE 3(7): e2593. doi:10.1371/journal.pone.0002593

