# Correction: Systemic Complement Activation in Age-Related Macular Degeneration

**DOI:** 10.1371/annotation/511a1029-bc43-4510-a4ca-c1db31810acc

**Published:** 2008-07-11

**Authors:** Hendrik P. N. Scholl, Peter Charbel Issa, Maja Walier, Stefanie Janzer, Beatrix Pollok-Kopp, Florian Börncke, Lars G. Fritsche, Ngaihang V. Chong, Rolf Fimmers, Thomas Wienker, Frank G. Holz, Bernhard H. F. Weber, Martin Oppermann

The Table S3 link leads to an incorrect file. Please view the correct Table S3 here:
