# Supplementary material for: Systemic Complement Activation in Age-Related Macular Degeneration
Source: PLoS One. 2008 Jul 2;3(7):e2593. doi: 10.1371/journal.pone.0002593 (PMC2440421; doi:10.1371/journal.pone.0002593)
Supplement: Table S1 — Genotypes (0.09 MB PDF) [file pone.0002593.s001.pdf]

SUPPL. TABLE 1. GENOTYPES Marker	Designation (Gene)	Allele 1	Allele 2	Genotypes Cases			MAF *	Genotypes Controls			MAF*	ATT **
				11	12	22		11	12	22		P value
**CFH gene**												
rs800292	I62V (CFH)	G	A	84<br>75.0%	27<br>24.1%	1<br>8.9%	12.9%	38<br>56.7%	27<br>40.3%	2<br>3.0%	23.1%	0.009
rs1061170	Y402H (CFH)	T	C	20<br>17.9%	56<br>50.0%	36<br>32.1%	57.1%	27<br>40.3%	32<br>47.8%	8<br>11.9%	35.8%	<0.001
rs1048663	IVS 9 (CFH)	G	A	78<br>69.6%	32<br>28.6%	2<br>1.8%	16.1%	47<br>70.1%	16<br>23.9%	4<br>6.0%	17.9%	0.66
rs2274700	A473A (CFH)	G	A	71<br>63.3%	35<br>31.2%	6<br>5.4%	16.5%	21<br>31.3%	36<br>53.7%	10<br>14.9%	41.8%	<0.001
rs412852	IVS 15 (CFH)	A	G	16<br>14.3%	53<br>47.3%	43<br>38.4%	62.1%	23<br>34.3%	34<br>50.7%	10<br>14.9%	40.3%	<0.001
rs11582939	IVS 18 (CFH)	C	T	75<br>67.0%	35<br>31.2%	2<br>1.8%	17.4%	46<br>68.7%	17<br>25.4%	4<br>6.0%	18.7%	0.76
**BF-C2 gene**												
rs9332739	E318D (C2)	G	C	105<br>93.8%	7<br>6.3%	0<br>0.0%	3.1%	62<br>92.5%	5<br>7.5%	0<br>0.0%	3.7%	0.75
rs547154	IVS 10 (C2)	G	T	106<br>94.6%	6<br>5.4%	0<br>0.0%	2.7%	57<br>85.1%	10<br>14.9%	0<br>0.0%	7.5%	0.03
rs4151667	L9H (CFB)	T	A	105<br>93.8%	7<br>6.3%	0<br>0.0%	3.1%	62<br>92.5%	5<br>7.5%	0<br>0.0%	3.7%	0.75
rs12614	R32W (CFB)	C	T	88<br>78.6%	23<br>20.5%	1<br>0.9%	11.2%	60<br>89.6%	7<br>10.4%	0<br>0.0%	5.2%	0.05
rs641153	R32Q (CFB)	G	A	106<br>94.6%	6<br>5.4%	0<br>0.0%	2.7%	57<br>85.1%	10<br>14.9%	0<br>0.0%	7.5%	0.03
**C3 gene**												
rs2230199	R102G (C3)	G	C	68<br>60.7%	35<br>31.2%	9<br>8.0%	23.7%	50<br>74.6%	16<br>23.9%	1<br>1.5%	13.4%	0.03

* MAF, minor allele frequency; ** ATT, Armitage's trend test
