# Supplementary material for: Systemic Complement Activation in Age-Related Macular Degeneration
Source: PLoS One. 2008 Jul 2;3(7):e2593. doi: 10.1371/journal.pone.0002593 (PMC2440421; doi:10.1371/journal.pone.0002593)
Supplement: Table S2 — Haplotypes (0.08 MB PDF) [file pone.0002593.s002.pdf]

SUPPLEMENTARY TABLE 2.
HAPLOTYPES AND THEIR FREQUENCIES IN THE CASE AND CONTROL POPULATION ESTIMATED BY FAMHAP.

Haplotype	Frequency	
	Cases (n = 112)	Controls (n = 67)
**CFH gene**		
A T G A A C	12.0%	23.1%
**G C G G G C**	56.7%	35.8%
G T A G A T	16.1%	17.9%
G T G A A C	8.1%	17.9%
G T G G G C	4.5%	4.5%
others	2.7%	0.7%
**BF-C2 gene**		
C G A C G	2.8%	2.4%
C T A C A	0.0%	1.3%
G G T C G	83.4%	85.3%
**G G T T G**	11.2%	4.8%
G T T C A	2.3%	5.7%
others	0.4%	0.4%
