# Supplementary material for: Correction: Systemic Complement Activation in Age-Related Macular Degeneration
Source: PLoS One. 2008 Jul 11;3(7):10.1371/annotation/511a1029-bc43-4510-a4ca-c1db31810acc. doi: 10.1371/annotation/511a1029-bc43-4510-a4ca-c1db31810acc (PMC2656691; doi:10.1371/annotation/511a1029-bc43-4510-a4ca-c1db31810acc)
Supplement: Supplementary file 1 [file pone.511a1029-bc43-4510-a4ca-c1db31810acc.s001.pdf]

Supplementary Table 3: Retrospective Power Analysis

Marker	Designation (Gene)	Risk Allele Frequency	Heterozygotes Relative Risk	Homozygotes Relative Risk	Power
**CFH gene**					
rs800292	I62V (CFH)	0.769	2.00	4.42	0.942
rs1061170	Y402H (CFH)	0.358	2.36	6.08	0.999
rs1048663	IVS 9 (CFH)	0.821	4.00	3.32	0.050
rs2274700	A473A (CFH)	0.582	1.62	5.63	0.999
rs412852	IVS 15 (CFH)	0.403	2.24	6.18	0.999
rs11582939	IVS 18 (CFH)	0.813	4.12	3.26	0.053
**CFB-C2 gene**					
rs9332739	E318D (C2)	0.963	1.21	2.42 *	0.264
rs547154	IVS 10 (C2)	0.925	3.10	6.20 *	0.488
rs4151667	L9H (CFB)	0.963	1.21	2.42 *	0.264
rs12614	R32W (CFB)	0.052	2.24	4.48 *	0.716
rs641153	R32Q (CFB)	0.925	3.10	6.20 *	0.488
**C3 gene**					
rs2230199	R102G (C3)	0.134	1.61	6.62	0.723

* Because the homozygous risk genotype was not observed, the homozygous relative risk could not be calculated, and was therefore estimated as two times the risk of the heterozygotes.

The power calculations were performed for a two-sided level of $\alpha = 0.05$ and based on the prevalence of AMD according to van Leeuwen R, Klaver CC, Vingerling JR, Hofman A, de Jong PT (2003) Epidemiology of age-related maculopathy: a review. Eur J Epidemiol 18: 845-854.
